# Cross-Domain OTFS Detection via Delay–Doppler Decoupling: Reduced-Complexity Design and Performance Analysis †

**DOI:** 10.3390/e27101062

**Published:** 2025-10-13

**Authors:** Mengmeng Liu, Shuangyang Li, Baoming Bai, Giuseppe Caire

**Affiliations:** 1 State Key Laboratory of ISN, Xidian University, Xi’an 710071, China; mmengliu@stu.xidian.edu.cn; 2 Electrical Engineering and Computer Science Department, Technische Universität Berlin, 10587 Berlin, Germany; shuangyang.li@tu-berlin.de (S.L.); caire@tu-berlin.de (G.C.)

**Keywords:** OTFS, iterative detection, LMMSE, treating interference as noise strategy, genie-aided strategy

## Abstract

In this paper, a reduced-complexity cross-domain iterative detection for orthogonal time frequency space (OTFS) modulation is proposed that exploits channel properties in both time and delay–Doppler domains. Specifically, we first show that in the time-domain effective channel, the path delay only introduces interference among samples in adjacent time slots, while the Doppler becomes a phase term that does not affect the channel sparsity. This investigation indicates that the effects of delay and Doppler can be decoupled and treated separately. This “band-limited” matrix structure further motivates us to apply a reduced-size linear minimum mean square error (LMMSE) filter to eliminate the effect of delay in the time domain, while exploiting the cross-domain iteration for minimizing the effect of Doppler by noticing that the time and Doppler are a Fourier dual pair. Furthermore, we apply eigenvalue decomposition to the reduced-size LMMSE estimator, which makes the computational complexity independent of the number of cross-domain iterations, thus significantly reducing the computational complexity. The bias evolution and variance evolution are derived to evaluate the average MSE performance of the proposed scheme, which shows that the proposed estimators suffer from only negligible estimation bias in both time and DD domains. Particularly, the state (MSE) evolution is compared with bounds to verify the effectiveness of the proposed scheme. Simulation results demonstrate that the proposed scheme achieves almost the same error performance as the optimal detection, but only requires a reduced complexity.

## 1. Introduction

Future wireless networks are envisioned to accommodate many emerging applications, such as low-earth orbit (LEO) satellites and unmanned aerial vehicles (UAVs), where the signal is inevitably transmitted over complex and challenging high-mobility channel scenarios [1,2]. In such scenarios, both time dispersion and frequency dispersion occur simultaneously. The popular orthogonal frequency division multiplexing (OFDM), known for its ability to combat time dispersion well, suffers from severe performance degradation due to inter-carrier interference (ICI) and carrier frequency offset (CFO) caused by Doppler shifts [3].

The recently proposed orthogonal time frequency space (OTFS) modulation has shown to be a good solution to signal transmissions over such challenging high-mobility channels [4,5]. Unlike OFDM, which modulates information symbols in the time–frequency (TF) domain, the information symbols in OTFS systems are multiplexed in the delay–Doppler (DD) domain, leading to the full exploration of appealing DD domain channel properties, including quasi-static, separable, and sparse properties [6,7], which in return facilitates the design of channel estimation and equalization. More importantly, OTFS can potentially achieve full channel diversity [8,9,10,11], which ensures better performance robustness compared to currently deployed OFDM over challenging transmission scenarios.

The promising performance of OTFS relies on advanced equalization to handle the DD domain interference caused by multipath transmissions. Conventional linear equalizers, such as zero forcing (ZF) and minimum mean square error (MMSE), are widely applied in OTFS due to their simple implementation. Considering the high complexity order of traditional matrix inversion operations in linear equalization, several works of literature have proposed low-complexity implementation of ZF/MMSE for different cases of OTFS systems, such as [12,13,14,15,16]. However, linear equalization cannot achieve optimal performance. As a classical nonlinear equalization scheme, maximum-likelihood sequence estimation (MLSE) is optimal. However, it usually requires prohibitively high detection complexity and cannot be directly applied to practical systems. Thus, the design of reduced-complexity detection for OTFS has acquired much attention. For instance, a message passing (MP) algorithm based on maximum a posteriori probability (MAP) was proposed in [17], where the DD domain inter-symbol interference (ISI) is Gaussian-approximated to reduce detection complexity. Furthermore, damping was introduced to improve the convergence. Nevertheless, the convergence performance is still limited by the 4-cycles in the factor graph (FG). In order to address it, an improved MP detector for OTFS that constructs an FG of girth 6 and then directly applies an exact sum-product algorithm (SPA) with linear complexity in the symbol constellation size was proposed in [18]. Furthermore, many improved detection algorithms based on MP were proposed, such as hybrid MAP and parallel interference cancellation [19] and Gaussian approximate MP [20]. In particular, a variational Bayes framework for OTFS detection that effectively mitigates the performance degradation caused by the short cycles of the probabilistic graphical model in the MP algorithm was introduced in [21].

Note that most OTFS detection schemes, including the aforementioned ones, operate in the DD domain. However, when the time and frequency resources for OTFS are limited, the DD domain channel matrix could be dense due to insufficient resolution of delay and Doppler, and, consequently, DD domain detection may suffer from high detection complexity [22]. As a special type of MP algorithm with a lower complexity, unitary approximate message passing (UAMP) was proposed in [23], which can achieve a promising error performance with an efficient implementation in the case of rich scattering environments or insufficient Doppler resolution by exploiting the circulant or sparsity structures of the channel matrix. In order to obtain the special structures, ideal bi-orthogonal waveforms are assumed to be used. In the case of more practical waveforms, null symbols or cyclic prefixes (CPs) are frequently inserted into the OTFS signal block, which leads to significant overhead. Due to the limitations of single-domain detection, many studies have conducted research on joint multi-domain detectors. The cross-domain iterative detection proposed in [24] was a preliminary attempt to solve this issue by considering the detection in both time and DD domains via iterative processing, which can achieve almost the same error performance as ML detection even in the presence of fractional Doppler shifts. The cross-domain iterative detection is motivated by the unitary transformation between the time and DD domains, ensuring that the detection error in one domain is principally orthogonal to that in the other domain. Thus, it allows for cross-domain iterations for signal detection without introducing error propagation. Subsequently, ref. [25] systematically analyzed three cross-domain iterative detection models between the frequency domain and the DD domain. Their work further evaluated the convergence behavior, computational complexity, and error-rate performance of these models, thereby providing valuable theoretical guidance for the design of cross-domain detection schemes. However, in these cross-domain iterative detection algorithms, a full-size linear minimum mean square error (LMMSE) filter is adopted in the time domain, which, as we will show later, does not fully exploit the advantages of cross-domain iteration.

In this paper, we propose a novel cross-domain iterative detection for OTFS with reduced complexity. The major motivation is that the effects of delay and Doppler can be decoupled and thus can be treated separately. In particular, the rationale behind our work is that the path delay only introduces interference among samples in adjacent time slots, while the Doppler behaves as a phase term that does not affect the sparsity of the time-domain OTFS effective channel. Consequently, the time-domain channel matrix has a “band-limited” structure. Based on this, we propose a reduced-size estimator in the time domain to eliminate the effect of delay, while relying on cross-domain iterations to minimize the effect of Doppler. Such an iterative scheme is motivated by the fact that time and Doppler are a Fourier dual pair and therefore the effect of Doppler can be minimized by iteratively exchanging the extrinsic information between the time and DD domains. Furthermore, we provide a detailed performance analysis for the proposed scheme by studying the bias evolution and the state evolution, and compare it with the boundary to further prove the effectiveness of the proposed scheme. The detection complexity is also discussed. The main contributions of this paper are summarized as follows.

- We derive the time domain and DD domain vectorized input–output relations for OTFS transmissions using the discrete Zak transform (DZT). Based on this, we analyze the properties of the time-domain effective channel matrix and reveal that the effects of delay and Doppler can be decoupled and treated separately. Furthermore, we propose a reduced-complexity cross-domain iterative detection that applies a reduced-size LMMSE in the time domain and a simple symbol-by-symbol detection in the DD domain and iteratively exchanges the extrinsic information via the unitary transformation. In particular, we further apply eigenvalue decomposition (EVD) to the proposed reduced-size LMMSE estimator, which makes the computational complexity independent of the number of cross-domain iterations, thus significantly reducing the complexity.
- We derive the average MSE of the proposed algorithm, which shows that the error performance of the estimator is characterized by the covariance of the observation and the bias of the estimator. We show that the proposed estimators suffer from only negligible estimation bias in both time and DD domains. We further derive the state evolution for the unbiased estimation case and the theoretical performance bounds under treating-the-interference-as-noise (TIN) and genie-aided strategies. More importantly, we show that the TIN bound and genie-aided bound converge to each other after a sufficient number of cross-domain iterations. The proposed reduced-size LMMSE estimator also aligns well with the mechanism of the cross-domain iterative detection.
- We investigate the converged error performance of the proposed algorithm by focusing on the effective DD domain signal-to-noise ratio (SNR) under the TIN and genie-aided strategies. We show that the upper bound of the effective SNR under the TIN strategy converges to that under the genie-aided strategy with the iteration of the cross-domain detection. Furthermore, we show that the effective SNR of the proposed algorithm can theoretically approach the maximum receiver SNR with a sufficient number of iterations for a given fading channel. This also demonstrates that the proposed algorithm can achieve near-optimal performance with sufficient iterations.
- We evaluate the MSE performance and the error performance of the proposed algorithm by numerical simulations. The numerical results agree with our analysis and demonstrate a near-optimal error performance.

Notations: $F_{N}$ and $F_{N}^{H}$ denote the discrete Fourier transform (DFT) matrix and inverse DFT (IDFT) matrix of size N×N, respectively; $I_{N}$ denotes the identity matrix of size N×N; ⊗ denotes the Kronecker product operator; ${\left(\cdot \right)}^{H}$ denotes the Hermitian transpose; ${\left(\cdot \right)}^{T}$ denotes the transpose; ${\left(\cdot \right)}^{*}$ denotes conjugation; $\mathrm{diag}\left\{\cdot \right\}$ denotes the diagonal matrix; $\delta \left(\cdot \right)$ is the Dirac delta function.

## 2. System Model

### 2.1. Backgrounds on OTFS Transmissions

**Figure 1:**
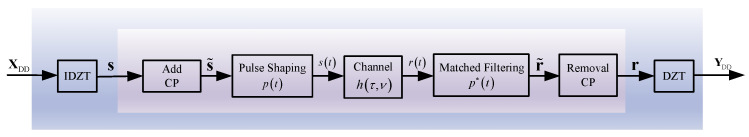
The transceiver structure of DZT-based OTFS transmissions.

Without loss of generality, let us consider the transceiver structure of OTFS transmissions using the discrete Zak transform (DZT) in Figure 1. Let *M* be the number of delay bins (sub-carriers) and *N* be the number of Doppler bins (time slots). Let *T* be the duration of each time slot and, correspondingly, the sub-carrier spacing is 1/T. A length-MN DD domain information symbol vector x, selected uniformly from a constellation alphabet $A=\left\{a_{1},\ldots ,a_{Q}\right\}$, is passed through the inverse DZT (IDZT) module, resulting in the time-domain transmitted signal s, i.e.,

(1) $$s=\left(F_{N}^{H}⊗I_{M}\right)x.$$

By applying the reduced-cyclic-prefix (reduced-CP) structure [26], the resultant time domain transmitted signal can be represented as

(2) $$\tilde{s}=A_{\mathrm{CP}}s,$$

where $A_{\mathrm{CP}}={\left[\begin{array}{cc} G_{\mathrm{CP}} & I_{MN} \end{array}\right]}^{T}$ is the block-wise CP addition matrix with size $\left(MN+L_{\mathrm{CP}}\right)\times MN$, and $G_{\mathrm{CP}}$ of size $MN\times L_{\mathrm{CP}}$ consists of the last $L_{\mathrm{CP}}$ columns of the identity matrix $I_{MN}$. Without loss of generality, the length of CPs, $L_{\mathrm{CP}}$, is selected to be no less than the maximum delay index, which will be introduced later. After adding CPs, the symbol $\tilde{s}\left[n\right]$ is transmitted by the $T_{s}$-orthogonal shaping pulse $p\left(t\right)$, yielding

(3) $$s\left(t\right)=\sum_{n=0}^{MN+L_{\mathrm{CP}}-1}\tilde{s}\left[n\right]p\left(t-nT_{s}\right).$$

Consider a path-*P* linear time-varying (LTV) wireless channel given by

(4) $$h\left(\tau ,\nu \right)=\sum_{i=1}^{P}h_{i}\delta \left(\tau -\tau_{i}\right)\delta \left(\nu -\nu_{i}\right),$$

where $h_{i}$ is the fading coefficient for the *i*-th path, following a complex Gaussian distribution with zero mean and variance 112P2P per real dimension (uniform power profile), and $\tau_{i}\in \left[0,T\right)$ and $\nu_{i}\in \left[0,1/T\right)$ represent the delay and Doppler shifts associated with the *i*-th path, respectively. In particular, we consider the discretized delay and Doppler indices defined by $l_{i}+\iota_{i}=\tau_{i}M/T$ and $k_{i}+\kappa_{i}=\nu_{i}NT$, where $0\leq l_{i}\leq M-1$ and $0\leq k_{i}\leq N-1$ are the corresponding integer indices of delay and Doppler for the *i*-th path, while $\iota_{i}\in \left(-0.5,\;0.5\right]$ and $\kappa_{i}\in \left(-0.5,\;0.5\right]$ represent the fractional contribution of the delay shift and the Doppler shift, respectively.

The transmitted time-domain signal $s\left(t\right)$ passes through the above LTV channel. At the receiver, the corresponding received time-domain signal $r\left(t\right)$ can be given by

(5) $$\begin{array}{cc} r\left(t\right) & =\;\int \int \;h\left(\tau ,\nu \right)s\left(t-\tau \right)e^{j2\pi \nu \left(t-\tau \right)}d\tau d\nu +n\left(t\right)\\ \; & =\sum_{i=1}^{P}\sum_{n=0}^{MN+L_{\mathrm{CP}}-1}h_{i}e^{j2\pi \nu_{i}\left(t-\tau_{i}\right)}\tilde{s}\left[n\right]p\left(t-nT_{s}-\tau_{i}\right)+n\left(t\right), \end{array}$$

where $n\left(t\right)$ is the additive white Gaussian noise (AWGN) process with the one-sided power spectral density (PSD) $N_{0}$. By applying a matched filter with the pulse $p^{*}\left(t\right)$ on $r\left(t\right)$, we can obtain

(6) $$\begin{array}{c} \tilde{r}\left[m\right]=\int_{-\infty }^{\infty }r\left(t\right)p^{*}\left(t-mT_{s}\right)dt=\sum_{i=1}^{P}\sum_{n=0}^{MN+L_{\mathrm{CP}}-1}g_{m,n}^{\left(i\right)}\tilde{s}\left[n\right]+\tilde{n}\left[m\right] \end{array}$$

for $m=0,\cdots ,MN+L_{\mathrm{CP}}-1$, where $g_{m,n}^{\left(i\right)}$ denotes the effective time-domain channel coefficient between the *n*-th transmitted symbol $\tilde{s}\left[n\right]$ and the *m*-th received symbol $\tilde{r}\left[m\right]$ over the *i*-th resolvable path. In (6), the coefficient $g_{m,n}^{\left(i\right)}$ can be expressed as

(7) $$g_{m,n}^{\left(i\right)}\overset{\Delta }{=}h_{i}e^{j2\pi n\nu_{i}T_{s}}A_{p}^{*}\left(\left(n-m\right)T_{s}+\tau_{i},\nu_{i}\right),$$

where $A_{p}\left(\tau ,\nu \right)$ denotes the ambiguity function of the pulse $p\left(t\right)$ with respect to delay τ and Doppler ν, given by

(8) $$A_{p}\left(\tau ,\nu \right)\overset{\Delta }{=}\int_{-\infty }^{\infty }p\left(t\right)p^{*}\left(t-\tau \right)e^{-j2\pi \nu \left(t-\tau \right)}dt.$$

Arrange (6) into a length-$\left(MN+L_{\mathrm{CP}}\right)$ vector $\tilde{r}$, i.e.,

(9) $$\tilde{r}=\sum_{i=1}^{P}G^{\left(i\right)}\tilde{s}+\tilde{n},$$

we define $G^{\left(i\right)}$ of size $\left(MN+L_{\mathrm{CP}}\right)\times \left(MN+L_{\mathrm{CP}}\right)$ as the time-domain effective channel matrix for the *i*-th resolvable path, whose $\left(m,n\right)$-th element is $g_{m,n}^{\left(i\right)}$. After performing CP removal with a block-wise CP removal matrix $R_{\mathrm{CP}}$ composed of the last MN rows of the identity matrix $I_{MN+L_{\mathrm{CP}}}$, the time-domain vectorized input–output relation for OTFS transmission can be given by

(10) $$r=\sum_{i=1}^{P}R_{\mathrm{CP}}G^{\left(i\right)}A_{\mathrm{CP}}s+R_{\mathrm{CP}}\tilde{n},$$

where $n=R_{\mathrm{CP}}\tilde{n}$ denotes the time-domain effective noise vector with zero mean and a one-sided power spectral density of $N_{0}$. Finally, the DD domain received symbol vector y can be obtained by performing DZT on r, written as

(11) $$y=\sum_{i=1}^{P}\left(F_{N}⊗I_{M}\right)H_{T}^{i}\left(F_{N}^{H}⊗I_{M}\right)x+\left(F_{N}⊗I_{M}\right)R_{\mathrm{CP}}\tilde{n},$$

where $H_{T}^{i}=R_{\mathrm{CP}}G^{\left(i\right)}A_{\mathrm{CP}}$ is the time-domain effective channel matrix for the *i*-th resolvable path.

### 2.2. Properties of the Time-Domain Effective Channel Matrix

**Figure 2:**
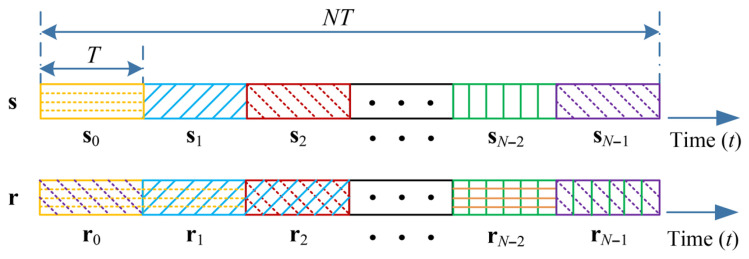
Brief illustration of the interference pattern between the time-domain transmitted vector s and the received vector r.

According to (10), the time-domain effective channel matrix $H_{T}$ after adding and removing CPs can be characterized as

(12) $$H_{T}=\sum_{i=1}^{P}R_{\mathrm{CP}}G^{\left(i\right)}A_{\mathrm{CP}},$$

whose size is MN×MN. As shown in (7), the path delay introduces the time-domain ISI, where the length of ISI is defined as $L_{\mathrm{ISI}}$. Noticing that $0\leq l_{p}\leq M-1$ and $-0.5<\iota_{i}\leq 0.5$, the effective maximum length of ISI is less than *M*. Furthermore, it should be pointed out that the maximum interference length can still be approximately limited to M−1 to consider fractional delay, since the interference outside the maximum interference length is so weak as to be almost negligible. Thus, it is clear that the interference induced by path delay is restricted to adjacent time slots [27]. Following the conventional OFDM setup, we partition s and r into *N* sub-blocks, each containing *M* samples, i.e., $r={\left[r_{0}^{H},r_{1}^{H},\ldots ,r_{N-1}^{H}\right]}^{H}$ and $s={\left[s_{0}^{H},s_{1}^{H},\ldots ,s_{N-1}^{H}\right]}^{H}$, respectively, as shown in Figure 2. Thus, the *i*-th received block $r_{i}$ can have interference from only the $\left(i-1\right)$-th block $s_{i-1}$ in the time domain. It should be noted that the first block $s_{0}$ is interfered with by the last block $s_{N-1}$ due to the appended reduced CP [28]. According to this sub-block structure, $H_{T}$ can be rewritten as

(13) $$H_{T}=\left[\begin{array}{cccccc} H_{T}^{0,0} & 0 & 0 & \ldots & 0 & H_{T}^{0,1}\\ H_{T}^{1,1} & H_{T}^{1,0} & 0 & \ldots & 0 & 0\\ 0 & H_{T}^{2,1} & H_{T}^{2,0} & \ldots & 0 & 0\\ \vdots & \vdots & \ddots & \ddots & \vdots & \vdots \\ 0 & 0 & 0 & \ldots & H_{T}^{N-2,0} & 0\\ 0 & 0 & 0 & \ldots & H_{T}^{N-1,1} & H_{T}^{N-1,0} \end{array}\right],$$

where $H_{T}^{i,0}$, i=0,…,N−1, of size M×M are the diagonal blocks of $H_{T}$, and $H_{T}^{i,1}$, i=0,…,N−1, of size M×M are the first sub-diagonal blocks of $H_{T}$, representing the inter-block interference from the $\left(i-1\right)$-th transmitted block to the *i*-th received block [28].

Furthermore, we notice that the Doppler effect behaves like a phase term in $H_{T}$, which does not affect the channel sparsity. The above observation suggests that the time-domain effective channel matrix $H_{T}$ has a “band-limited" structure, where each row only has limited non-zero elements.

According to the above analysis, we reformulate the block-wise input–output relation in the time domain as

(14) $$r_{i}=H_{T}^{i,0}s_{i}+H_{T}^{i,1}s_{{\left(i-1\right)}_{N}}+n_{i},$$

i=0,…,N−1, where ${\left(\cdot \right)}_{N}$ denotes mod-*N* operation. Note that both $r_{i}$ and $r_{{\left(i+1\right)}_{N}}$ contain the information of $s_{i}$ due to the path delay. Thus, it is convenient to write

(15) $$\begin{array}{cc} \left[\begin{array}{c} r_{i}\\ r_{{\left(i+1\right)}_{N}} \end{array}\right] & =\left[\begin{array}{c} H_{T}^{i,0}\\ H_{T}^{{\left(i+1\right)}_{N},1} \end{array}\right]s_{i}+\left[\begin{array}{cc} H_{T}^{i,1} & \\ & H_{T}^{{\left(i+1\right)}_{N},0} \end{array}\right]\left[\;\;\begin{array}{c} s_{{\left(i-1\right)}_{N}}\\ s_{{\left(i+1\right)}_{N}} \end{array}\right]+\left[\begin{array}{c} n_{i}\\ n_{{\left(i+1\right)}_{N}} \end{array}\right], \end{array}$$

which can be further written as

(16) $${\tilde{r}}_{i}=H_{A}^{i}s_{i}+H_{B}^{i}{\tilde{s}}_{i}+{\tilde{n}}_{i}.$$

In (16), ${\tilde{r}}_{i}\in C^{2M\times 1}$ is the observation at the receiver side corresponding to $s_{i}$; $H_{A}^{i}\in C^{2M\times M}$ is the effective observation matrix, characterizing the interference pattern related to $s_{i}$; $H_{B}^{i}\in C^{2M\times 2M}$ is the effective interference matrix, characterizing the additional interference from other transmitted sub-blocks; ${\tilde{s}}_{i}\in C^{2M\times 1}$ is the interfering signal vector; and ${\tilde{n}}_{i}\in C^{2M\times 1}$ is the considered noise vector.

The above discussion naturally motivates us to design a time-domain estimator/detector that exploits the effective channel matrix structure, and this is presented in the following section.

## 3. Cross-Domain Iterative Detection for OTFS Modulation via Delay–Doppler Decoupling

Based on (16), we propose applying a reduced-size LMMSE estimator for eliminating the delay interference that has a much lower complexity compared to the full-size LMMSE estimator adopted in [24]. However, such an estimator cannot fully minimize the effect of Doppler. In [24], the authors have shown that the cross-domain iteration can effectively detect the OTFS signal by iteratively exchanging the extrinsic information between the time domain and the DD domain due to the fact that time and Doppler are a Fourier dual pair. Following this idea, we adopt the cross-domain iteration to minimize the Doppler interference as will be discussed later. For clarity, we assume perfect knowledge of channel state information (CSI), including the number of resolvable paths, path delays, Doppler shifts, and fading coefficients. In practice, the DD domain channel remains roughly constant within a stationarity region [7,11,28]. Consequently, acquiring CSI in the DD domain is generally feasible, even in high-mobility scenarios.

### 3.1. Reduced-Size LMMSE Estimator in the Time Domain

Based on (16), the block-wise LMMSE estimation matrix $W_{\mathrm{MMSE}}^{i}$ for the *i*-th transmitted block $s_{i}$ can be obtained as

(17) $$\begin{array}{cc} W_{\mathrm{MMSE}}^{i} & =C_{s_{i}}^{a,T}{\left(H_{A}^{i}\right)}^{H}\left(H_{A}^{i}C_{s_{i}}^{a,T}{\left(H_{A}^{i}\right)}^{H}\right.{\left.+H_{B}^{i}C_{{\tilde{s}}_{i}}^{a,T}{\left(H_{B}^{i}\right)}^{H}+N_{0}I_{2M}\right)}^{-1}, \end{array}$$

where $C_{s_{i}}^{a,T}$ and $C_{{\tilde{s}}_{i}}^{a,T}$ are the a priori covariance matrices of $s_{i}$ and ${\tilde{s}}_{i}$ and initialized as $I_{M}$ and $I_{2M}$ for the first iteration, respectively. Furthermore, the a posteriori estimation output $m_{s_{i}}^{p,T}$ of $s_{i}$ is given by

(18) $$m_{s_{i}}^{p,T}=m_{s_{i}}^{a,T}+W_{\mathrm{MMSE}}^{i}\left({\tilde{r}}_{i}-H_{B}^{i}m_{{\tilde{s}}_{i}}^{a,T}-H_{A}^{i}m_{s_{i}}^{a,T}\right),$$

where $m_{s_{i}}^{a,T}\;$ and $m_{{\tilde{s}}_{i}}^{a,T}\;$ are the a priori mean vectors of $s_{i}\;$ and ${\tilde{s}}_{i}$ with sizes M×1 and 2M×1, respectively. Note that the above LMMSE estimator applies the successive interference cancellation (SIC) to eliminate the interference from ${\tilde{s}}_{i}$ according to the a priori information from the previous iteration.

The a posteriori covariance matrix $C_{s_{i}}^{p,T}$ of $s_{i}$ is given by

(19) $$C_{s_{i}}^{p,T}=C_{s_{i}}^{a,T}-W_{\mathrm{MMSE}}^{i}H_{A}^{i}C_{s_{i}}^{a,T}.$$

Note that $C_{s_{i}}^{a,T}\;\;$ should be a diagonal matrix due to the independent and identically distributed (i.i.d.) assumption of the transmitted symbols, which is independent of channel impairments such as fractional Doppler shifts and waveform distortions. As such, we discard the non-diagonal entries of $C_{s_{i}}^{p,T}$ (treated as zeros) for further processing [24]. The extrinsic covariance matrix $C_{s_{i}}^{e,T}$ and mean $m_{s_{i}}^{e,T}$ associated with $s_{i}$ from the LMMSE estimation can be written as

(20) $$C_{s_{i}}^{e,T}={\left({\left(C_{s_{i}}^{p,T}\right)}^{-1}-{\left(C_{s_{i}}^{a,T}\right)}^{-1}\right)}^{-1}$$

and

(21) $$m_{s_{i}}^{e,T}=C_{s_{i}}^{e,T}\left({\left(C_{s_{i}}^{p,T}\right)}^{-1}m_{s_{i}}^{p,T}-{\left(C_{s_{i}}^{a,T}\right)}^{-1}m_{s_{i}}^{a,T}\right),$$

respectively.

According to (17), it is clear that the computational complexity depends on the inverse operation, which is in the order of $O\left({\left(2M\right)}^{3}\right)$. Since *N* sub-blocks need to be detected, the overall computational complexity of the proposed reduced-size LMMSE estimator per execution is $O\left({\left(2M\right)}^{3}N\right)$. In (13), we consider a one-sided ISI of length $L_{\mathrm{ISI}}$, since the path delay is not less than zero. Thus, $H_{T}^{i,0}$ is a lower triangular matrix, while $H_{T}^{i,1}$ is a strictly upper triangular matrix. Obviously, both $H_{A}^{i}C_{s_{i}}^{a,T}{\left(H_{A}^{i}\right)}^{H}$ and $H_{B}^{i}C_{{\tilde{s}}_{i}}^{a,T}{\left(H_{B}^{i}\right)}^{H}$ are banded matrices with halfwidth $L_{\mathrm{ISI}}+1$. Therefore, we can utilize LU decomposition [29] to further reduce the complexity of the inverse operation in (17) to $O\left(\left(2M\right)\left({\left(2L_{\mathrm{ISI}}+1\right)}^{2}+2\left(2L_{\mathrm{ISI}}+1\right)\right)\right)$. Nevertheless, when the ISI length is large, the computational complexity of the LU-based reduced-size LMMSE scheme approaches that of the reduced-size LMMSE scheme. Interestingly, by investigating (17), we find that only the a priori information $C_{s}^{a,T}$ of the proposed reduced-size LMMSE estimator is updated in each cross-domain iteration. Note that the diagonal entries of the diagonal matrix $C_{s}^{a,T}$ tend to be of the same value due to the law of large numbers, when MN tends to infinity. These observations motivate us to use the eigenvalue decomposition (EVD) to minimize the computation of (17) in each cross-domain iteration. We refer to this scheme as EVD-based reduced-size LMMSE, which will be described in detail in the following subsection.

### 3.2. EVD-Based Reduced-Size LMMSE Estimator in the Time Domain

We assume that the a priori covariance matrix $C_{s}^{a,T}$ is a diagonal matrix with the same diagonal entries converging to

(22) $$C_{s}^{a,T}=\frac{1}{MN}\mathrm{Tr}\left(C_{s}^{e,\mathrm{DD}}\right)I_{MN}=\alpha_{n_{\mathrm{iter}}}I_{MN},$$

which also verifies that such an assumption is aligned with the law of large numbers and only causes negligible performance loss when MN is sufficiently large, especially in the high-SNR regime [24]. Thus, substituting $C_{s_{i}}^{a,T}=\alpha_{n_{\mathrm{iter}}}I_{M}$ and $C_{{\tilde{s}}_{i}}^{a,T}=\alpha_{n_{\mathrm{iter}}}I_{2M}$ into (17), we can obtain

(23) $$W_{\mathrm{MMSE}}^{i}={\left(H_{A}^{i}\right)}^{H}{\left(H_{A}^{i}{\left(H_{A}^{i}\right)}^{H}+H_{B}^{i}{\left(H_{B}^{i}\right)}^{H}+\frac{N_{0}}{\alpha_{n_{iter}}}I_{2M}\right)}^{-1}.$$

Furthermore, we define

(24) $$\Phi^{i}={\left(H_{A}^{i}{\left(H_{A}^{i}\right)}^{H}+H_{B}^{i}{\left(H_{B}^{i}\right)}^{H}+\frac{N_{0}}{\alpha_{n_{iter}}}I_{2M}\right)}^{-1}$$

and

(25) $$\Psi^{i}=H_{A}^{i}{\left(H_{A}^{i}\right)}^{H}+H_{B}^{i}{\left(H_{B}^{i}\right)}^{H}.$$

Evidently, (25) is a Hermitian matrix with size 2M×2M, which can be decomposed by EVD into

(26) $$\Psi^{i}=U_{i}\Lambda_{i}U_{i}^{H}.$$

Specifically, $U_{i}$ with size 2M×2M is a unitary matrix whose *j*-th column is the eigenvector of $\Psi^{i}$, and $\Lambda_{i}$ is a diagonal matrix whose diagonal elements are the corresponding eigenvalues, i.e., $\Lambda_{i}=\mathrm{diag}\left\{\lambda_{1}^{i},\ldots ,\lambda_{j}^{i},\ldots ,\lambda_{2M}^{i}\right\}$. Consequently, (24) can be simplified as

(27) $$\Phi^{i}=U_{i}{\left(\Lambda_{i}+\frac{N_{0}}{\alpha_{n_{iter}}}I_{2M}\right)}^{-1}U_{i}^{H}=U_{i}{\tilde{\Lambda }}_{i}U_{i}^{H},$$

where

(28) $$\begin{array}{cc} {\tilde{\Lambda }}_{i} & ={\left(\Lambda_{i}+\frac{N_{0}}{\alpha_{n_{iter}}}I_{2M}\right)}^{-1} \end{array}$$

(29) $$\begin{array}{cc} \;\; & =\mathrm{diag}\left\{\frac{1}{\lambda_{1}^{i}+N_{0}/\alpha_{n_{\mathrm{iter}}}},\ldots ,\frac{1}{\lambda_{2M}^{i}+N_{0}/\alpha_{n_{\mathrm{iter}}}}\right\} \end{array}$$

is updated in each cross-domain iteration with negligible computational complexity compared to that of the matrix inversion operation.

The computational complexity of the proposed EVD-based reduced-size LMMSE estimator mainly depends on the EVD of (25), which is generally in the order of $O\left({\left(2M\right)}^{3}\right)$, consistent with that of the matrix inversion operation in the reduced-size LMMSE estimator. However, the EVD operation used in the proposed scheme only needs to be performed in the first iteration and then retained for subsequent cross-domain iterations. Thus, the computational complexity of the proposed EVD-based reduced-size LMMSE scheme does not become cumulatively larger as the number of cross-domain iteration increases, i.e., $O\left({\left(2M\right)}^{3}N\right)$, which significantly reduces the computational complexity of the entire cross-domain iteration detection.

### 3.3. Cross-Domain Iterative Detection for OTFS

**Figure 3:**
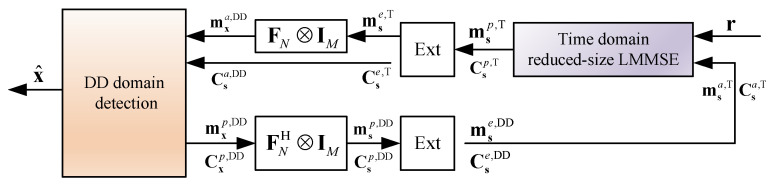
The block diagram of the considered cross-domain iterative receiver with the time-domain reduced-size LMMSE.

The extrinsic information $C_{s_{i}}^{e,T}$ and $m_{s_{i}}^{e,T}$ obtained from the LMMSE estimation is passed to the DD domain as shown in Figure 3. According to the relationship between the DD domain and time domain, the DD-domain a priori information, $C_{s}^{a,\mathrm{DD}}$ and $m_{x}^{a,\mathrm{DD}}$, is given by [24]

(30) $$C_{s}^{a,\mathrm{DD}}=C_{s}^{e,T},$$

and

(31) $$m_{x}^{a,\mathrm{DD}}=\left(F_{N}⊗I_{M}\right)m_{s}^{e,T},$$

where $C_{s}^{e,T}=\mathrm{diag}\left\{\mathrm{diag}\left\{C_{s_{0}}^{e,T}\right\},\ldots ,\mathrm{diag}\left\{C_{s_{N-1}}^{e,T}\right\}\right\}$ and $m_{s}^{e,T}\;\;=\;{\left[{\left(m_{s_{0}}^{e,T}\right)}^{T},\ldots ,{\left(m_{s_{N-1}}^{e,T}\right)}^{T}\right]}^{T}\;\;\;$ are the extrinsic covariance matrix and mean of s, respectively.

In the DD domain, the detection can be conducted in a simple symbol-by-symbol manner, e.g., Algorithm 2 in [24], where the corresponding a posteriori mean $m_{x}^{p,\mathrm{DD}}\;$ and covariance matrix $C_{x}^{p,\mathrm{DD}}\;$ of the DD domain OTFS symbol x are then passed back to the time domain for calculating the extrinsic information. Based on (1), the corresponding a posteriori mean $m_{s}^{p,\mathrm{DD}}$ and covariance matrix $C_{s}^{p,\mathrm{DD}}$ of s are given by

(32) $$m_{s}^{p,\mathrm{DD}}=\left(F_{N}^{H}⊗I_{M}\right)m_{x}^{p,\mathrm{DD}},$$

and

(33) $$C_{s}^{p,\mathrm{DD}}=\left(F_{N}^{H}⊗I_{M}\right)C_{x}^{p,\mathrm{DD}}\left(F_{N}⊗I_{M}\right),$$

respectively. Then, the extrinsic information of s in terms of the covariance matrix and mean can be obtained as [24]

(34) $$C_{s}^{e,\mathrm{DD}}={\left({\left(C_{s}^{p,\mathrm{DD}}\right)}^{-1}-{\left(C_{s}^{a,\mathrm{DD}}\right)}^{-1}\right)}^{-1},$$

and

(35) $$m_{s}^{e,\mathrm{DD}}\;=\;C_{s}^{e,\mathrm{DD}}\;\left(\;{\left(C_{s}^{p,\mathrm{DD}}\right)}^{\;-1}m_{s}^{p,\mathrm{DD}}\;-\;{\left(C_{s}^{a,\mathrm{DD}}\right)}^{\;-1}m_{s}^{e,T}\;\right),$$

respectively. Next, the extrinsic information is fed back to the time-domain LMMSE estimator for the coming iteration. Specifically, the a priori covariance matrix and mean of s are updated to $C_{s}^{a,T}=C_{s}^{e,\mathrm{DD}}$ and $m_{s}^{a,T}=m_{s}^{e,\mathrm{DD}}$. The details of the proposed low-complexity cross-domain iterative detection for OTFS are given in Algorithm 1.
**Algorithm 1** Low-Complexity Cross-Domain Iterative Detection for OTFS   1:**Input:** r, $H_{T}$, $N_{0}$, and maximum iteration $L_{\max}$   2:**Initialization:** Set $C_{s}^{a,T}=I_{MN}$, $m_{z}^{a,T}=0_{MN}$, $n_{\mathrm{iter}}=1$   3:**while** $n_{\mathrm{iter}}\leq L_{\max}$ **do**   4:    **for** i=0,1,…,N−1 **do**   5:       Compute the time domain LMMSE estimator matrix $W_{\mathrm{MMSE}}^{i}$ by (17) or (23)   6:       Compute the *a posteriori* information $m_{s_{i}}^{p,T}$ based on (18) and $C_{s_{i}}^{p,T}$ based on (19).   7:       Compute the extrinsic information $C_{s_{i}}^{e,T}$ based on (20) and $m_{s_{i}}^{e,T}$ based on (21).   8:   **end for**   9:   Compute the DD domain *a priori* information $C_{s}^{a,\mathrm{DD}}$ based on (30) and $m_{x}^{a,\mathrm{DD}}$ based on (31). 10:   Perform the symbol-by-symbol detection for DD domain symbols according to Algorithm 2 in [24]. 11:   Compute the *a posteriori* information of the time domain signal s, i.e., $m_{s}^{p,\mathrm{DD}}$ based on (32) and $C_{s}^{p,\mathrm{DD}}$ based on (33). 12:   Compute the extrinsic information $C_{s}^{e,\mathrm{DD}}$ based on (34) and $m_{s}^{e,\mathrm{DD}}$ based on (35). 13:   Refresh the time domain *a priori* information $C_{s}^{a,T}=C_{s}^{e,\mathrm{DD}}$ and $m_{s}^{a,T}=m_{s}^{e,\mathrm{DD}}$. 14:   $n_{\mathrm{iter}}=n_{\mathrm{iter}}+1$ 15:**end while** 16:**Output:** DD domain estimated signal $\hat{x}$.

## 4. Performance Analysis

In this section, we analyze the MSE performance and error performance of the proposed reduced-size cross-domain iterative detection algorithm.

### 4.1. Average MSE Analysis

Let us consider the a priori mean $m_{x}^{a,\mathrm{DD}}$ of the DD domain detector. Recalling (31), $m_{x}^{a,\mathrm{DD}}$ is related to the extrinsic mean output of the time-domain detector, i.e., $m_{s}^{e,T}$. We rewrite $m_{s_{i}}^{e,T}$ in (21) as follows:

(36) $$\begin{array}{cc} m_{s_{i}}^{e,T}\left[k\right] & =v_{s_{i}}^{e,T}\left[k\right]\left(\frac{m_{s_{i}}^{p,T}\left[k\right]}{v_{s_{i}}^{p,T}\left[k\right]}-\frac{m_{s_{i}}^{a,T}\left[k\right]}{v_{s_{i}}^{a,T}\left[k\right]}\right)\\ \; & =\frac{1}{\frac{1}{v_{s_{i}}^{p,T}\left[k\right]}-\frac{1}{v_{s_{i}}^{a,T}\left[k\right]}}\left(\frac{m_{s_{i}}^{p,T}\left[k\right]}{v_{s_{i}}^{p,T}\left[k\right]}-\frac{m_{s_{i}}^{a,T}\left(k\right)}{v_{s_{i}}^{a,T}\left[k\right]}\right)\\ \; & =\frac{m_{s_{i}}^{p,T}\left[k\right]v_{s_{i}}^{a,T}\left[k\right]-m_{s_{i}}^{a,T}\left[k\right]v_{s_{i}}^{p,T}\left[k\right]}{v_{s_{i}}^{a,T}\left[k\right]-v_{s_{i}}^{p,T}\left[k\right]}, \end{array}$$

with respect to the index *k*. In (36), $v_{s_{i}}^{e,T}\left[k\right]$, $v_{s_{i}}^{p,T}\left[k\right]$, and $v_{s_{i}}^{a,T}\left[k\right]$ represent the *k*-th diagonal element of $C_{s_{i}}^{e,T}$, $C_{s_{i}}^{p,T}$, and $C_{s_{i}}^{a,T}$, respectively. According to (18), we define

(37) $$m_{s_{i}}^{p,T}\left[k\right]=m_{s_{i}}^{a,T}\left[k\right]+{\tilde{m}}_{s_{i}}^{T}\left[k\right],$$

where ${\tilde{m}}_{s_{i}}^{T}\left[k\right]$ is the *k*-th element of the vector

(38) $${\tilde{m}}_{s_{i}}^{T}=W_{\mathrm{MMSE}}^{i}\left({\tilde{r}}_{i}-H_{B}^{i}m_{{\tilde{s}}_{i}}^{a,T}-H_{A}^{i}m_{s_{i}}^{a,T}\right).$$

Substituting (37) into (36), we further obtain

(39) $$\begin{array}{cc} m_{s_{i}}^{e,T}\left[k\right] & =\frac{\left(m_{s_{i}}^{a,T}\left[k\right]+{\tilde{m}}_{s_{i}}^{T}\left[k\right]\right)v_{s_{i}}^{a,T}\left[k\right]-m_{s_{i}}^{a,T}\left[k\right]v_{s_{i}}^{p,T}\left[k\right]}{v_{s_{i}}^{a,T}\left[k\right]-v_{s_{i}}^{p,T}\left[k\right]}\\ \; & =m_{s_{i}}^{a,T}\left[k\right]+\frac{v_{s_{i}}^{a,T}\left[k\right]}{v_{s_{i}}^{a,T}\left[k\right]-v_{s_{i}}^{p,T}\left[k\right]}{\tilde{m}}_{s_{i}}^{T}\left[k\right]. \end{array}$$

Vectorizing (39), we have

(40) $$m_{s_{i}}^{e,T}=m_{s_{i}}^{a,T}+{\tilde{C}}_{s_{i}}^{T}{\tilde{m}}_{s_{i}}^{T},$$

where ${\tilde{C}}_{s_{i}}^{T}\overset{\Delta }{=}\mathrm{diag}\left\{\frac{v_{s_{i}}^{a,T}\left[k\right]}{v_{s_{i}}^{a,T}\left[k\right]-v_{s_{i}}^{p,T}\left[k\right]}\right\}$ for k=0,…,M−1. Arranging $m_{s_{i}}^{e,T}$, i=0,…,N−1, into a length-MN vector $m_{s}^{e,T}$, we can obtain

(41) $$m_{s}^{e,T}=m_{s}^{a,T}+{\tilde{C}}_{s}^{T}{\tilde{m}}_{s}^{T},$$

where ${\tilde{C}}_{s}^{T}=\mathrm{diag}\left\{{\tilde{C}}_{s_{0}}^{T},\ldots ,{\tilde{C}}_{s_{N-1}}^{T}\right\}$ and ${\tilde{m}}_{s}^{T}={\left[{\left({\tilde{m}}_{s_{0}}^{T}\right)}^{T},\ldots ,{\left({\tilde{m}}_{s_{N-1}}^{T}\right)}^{T}\right]}^{T}$. Let us focus on the form of ${\tilde{m}}_{s}^{T}$, given by

(42) $${\tilde{m}}_{s}^{T}=WH_{A}s-WH_{A}m_{s}^{a,T}+WH_{B}\tilde{s}-WH_{B}m_{\tilde{s}}^{a,T}+W\tilde{n},$$

where $W=\mathrm{diag}\left\{W_{\mathrm{MMSE}}^{0},\ldots ,W_{\mathrm{MMSE}}^{N-1}\right\}$ with size MN×2MN, $H_{A}=\mathrm{diag}\left\{H_{A}^{0},\ldots ,H_{A}^{N-1}\right\}$ with size 2MN×MN, $H_{B}=\mathrm{diag}\left\{H_{B}^{0},\ldots ,H_{B}^{N-1}\right\}$ with size 2MN×2MN, $\tilde{s}={\left[{\tilde{s}}_{0}^{H},\ldots ,{\tilde{s}}_{N-1}^{H}\right]}^{H}$ with size 2MN×2MN, $m_{\tilde{s}}^{a,T}={\left[{\left(m_{{\tilde{s}}_{0}}^{a,T}\right)}^{H},\ldots ,{\left(m_{{\tilde{s}}_{N-1}}^{a,T}\right)}^{H}\right]}^{H}$ with size 2MN×1, and $\tilde{n}={\left[{\tilde{n}}_{0}^{H},\ldots ,{\tilde{n}}_{N-1}^{H}\right]}^{H}$ with size 2MN×1. According to (31), (38), and (42), we can rewrite $m_{x}^{a,\mathrm{DD}}$ as

(43) $$\begin{array}{cc} m_{x}^{a,\mathrm{DD}} & =\left(F_{N}⊗I_{M}\right)\left(m_{s}^{a,T}+{\tilde{C}}_{s}^{T}{\tilde{m}}_{s}^{T}\right)\\ \; & =\left(F_{N}⊗I_{M}\right)\left({\tilde{C}}_{s}^{T}WH_{A}s\right.+\left(I_{MN}-{\tilde{C}}_{s}^{T}WH_{A}\right)m_{s}^{a,T}+{\tilde{C}}_{s}^{T}WH_{B}\left(\tilde{s}-m_{\tilde{s}}^{a,T}\right)+\left.{\tilde{C}}_{s}^{T}W\tilde{n}\right)\\ \; & =x+\left(\left(F_{N}⊗I_{M}\right){\tilde{C}}_{s}^{T}WH_{A}\left(F_{N}^{H}⊗I_{M}\right)-I_{MN}\right)x+\left(F_{N}⊗I_{M}\right)\left(I_{MN}-{\tilde{C}}_{s}^{T}WH_{A}\right)m_{s}^{a,T}\\ \; & \;+\left(F_{N}⊗I_{M}\right){\tilde{C}}_{s}^{T}WH_{B}\left(\tilde{s}-m_{\tilde{s}}^{a,T}\right)+\left(F_{N}⊗I_{M}\right){\tilde{C}}_{s}^{T}W\tilde{n}\\ \; & =x+\left(\left(F_{N}⊗I_{M}\right){\tilde{C}}_{s}^{T}WH_{A}-\left(F_{N}⊗I_{M}\right)\right)s+\left(F_{N}⊗I_{M}\right)\left(I_{MN}-{\tilde{C}}_{s}^{T}WH_{A}\right)m_{s}^{a,T}\\ \; & \;+\left(F_{N}⊗I_{M}\right){\tilde{C}}_{s}^{T}WH_{B}\left(\tilde{s}-m_{\tilde{s}}^{a,T}\right)+\left(F_{N}⊗I_{M}\right){\tilde{C}}_{s}^{T}W\tilde{n} \end{array}$$

Investigating (43), we can obtain that the mean of $m_{x}^{a,\mathrm{DD}}$, i.e., $E\left[m_{x}^{a,\mathrm{DD}}\right]$, approaches zero when $E\left[m_{s}^{a,T}\right]=s$. From the derived condition for unbiased estimation in the DD domain, we note that it depends on the accuracy of the a priori mean $m_{x}^{a,\mathrm{DD}}$ of the time-domain estimator in the current *l*-th iteration, which is related not only to the output of the DD-domain estimator in the $\left(l-1\right)$-th iteration but also to the accuracy of the output of the DD domain in all previous iterations. Evidently, according to (35), $m_{s}^{a,T}\left(l\right)$ depends on $m_{s}^{e,T}\left(l-1\right)$, $C_{s}^{a,\mathrm{DD}}\left(l-1\right)$, $m_{s}^{p,\mathrm{DD}}\left(l-1\right)$, $m_{x}^{p,\mathrm{DD}}\left(l-1\right)$, $C_{s}^{p,\mathrm{DD}}\left(l-1\right)$ and $C_{x}^{p,\mathrm{DD}}\left(l-1\right)$, which are updated recursively in the iterations. Therefore, the quality of the proposed algorithm depends on the qualities of the time-domain and DD-domain estimators and can be improved by recursive iterations. This analysis will be further demonstrated in numerical results.

Without loss of generality, we will characterize the error performance of the proposed algorithm by tracking the average MSEs in both the time domain and the DD domain. We define the average MSEs of the inputs of the time-domain estimator and the DD-domain estimator in the *l*-th iteration as

(44) $${\mathrm{MSE}}_{T}\left(l\right)\overset{\Delta }{=}\frac{1}{MN}E\left[{\left(m_{s}^{a,T}\left(l\right)-s\right)}^{H}\left(m_{s}^{a,T}\left(l\right)-s\right)\right]$$

and

(45) $${\mathrm{MSE}}_{\mathrm{DD}}\left(l\right)\overset{\Delta }{=}\frac{1}{MN}E\left[{\left(m_{x}^{a,\mathrm{DD}}\left(l\right)-x\right)}^{H}\left(m_{x}^{a,\mathrm{DD}}\left(l\right)-x\right)\right],$$

respectively. Based on the properties of the expectation operation, (44) can be further derived as

(46) $$\begin{array}{cc} {\mathrm{MSE}}_{T}\left(l\right)\; & =\frac{1}{MN}E\left[{\left(m_{s}^{a,T}\left(l\right)-E\left[m_{s}^{a,T}\left(l\right)\right]+E\left[m_{s}^{a,T}\left(l\right)\right]-s\right)}^{H}\right.\\ \; & \;\left.\left(m_{s}^{a,T}\left(l\right)-E\left[m_{s}^{a,T}\left(l\right)\right]+E\left[m_{s}^{a,T}\left(l\right)\right]-s\right)\right]\\ \; & =\frac{1}{MN}E\left[{\left(m_{s}^{a,T}\left(l\right)-E\left[m_{s}^{a,T}\left(l\right)\right]\right)}^{H}\left(m_{s}^{a,T}\left(l\right)-E\left[m_{s}^{a,T}\left(l\right)\right]\right)\right]\\ \; & \;+\frac{1}{MN}E\left[{\left(E\left[m_{s}^{a,T}\left(l\right)\right]-s\right)}^{H}\left(E\left[m_{s}^{a,T}\left(l\right)\right]-s\right)\right]\\ \; & =\frac{1}{MN}\mathrm{Tr}\left(C_{m_{s}^{a,T}}\left(l\right)\right)+\frac{1}{MN}{∥\mathrm{bias}\left(m_{s}^{a,T}\left(l\right),s\right)∥}^{2}, \end{array}$$

where

(47) $$C_{m_{s}^{a,T}}\left(l\right)=E\left[\left(m_{s}^{a,T}\left(l\right)-E\left[m_{s}^{a,T}\left(l\right)\right]\right){\left(m_{s}^{a,T}\left(l\right)-E\left[m_{s}^{a,T}\left(l\right)\right]\right)}^{H}\right]$$

denotes the covariance matrix of $m_{s}^{a,T}\left(l\right)$, and $\mathrm{bias}\left(m_{s}^{a,T}\left(l\right),s\right)=E\left[m_{s}^{a,T}\left(l\right)\right]-s$ denotes the bias vector between $m_{s}^{a,T}\left(l\right)$ and s. Similar to (46), (45) can be reformulated as

(48) $${\mathrm{MSE}}_{\mathrm{DD}}\left(l\right)=\frac{1}{MN}\mathrm{Tr}\left(C_{m_{x}^{a,\mathrm{DD}}}\left(l\right)\right)+\frac{1}{MN}{∥\mathrm{bias}\left(m_{x}^{a,\mathrm{DD}}\left(l\right),x\right)∥}^{2}$$

Based on (46) and (48), the characterization of the error performance of the proposed algorithm is divided into two parts, namely the covariance of the observation of z (or x) and the bias of the observation of z (or x) and z (or x). In the following subsections, we will analyze in detail the impact of the covariance of the observation and the bias of the estimator on the error performance of the estimator, respectively.

### 4.2. Bias Analysis by Monte Carlo Method

In this subsection, we explore the evolution of the average biases of the time-domain and DD-domain estimators. By utilizing the Monte Carlo method, we recursively track the average biases of the two domains in each iteration, which can be described as follows. Firstly, we establish a given time-domain effective channel matrix, as shown in (12), and a given signal-to-noise ratio (SNR). Then, in each Monte Carlo trial, the DD-domain transmitted symbol x is regenerated and transmitted according to the system model described in Section 2.1. During the transmission process, the DD-domain transmitted signal x and the time-domain transmitted signal z are retained for subsequent bias calculation. At the receiver, the proposed algorithm is executed multiple times to obtain the mean of the observation, i.e., $E\left[m_{s}^{a,T}\right]$ and $E\left[m_{s}^{a,\mathrm{DD}}\right]$. Finally, the average bias performance can be obtained by performing sufficient Monte Carlo trials as described above.

**Figure 4:**
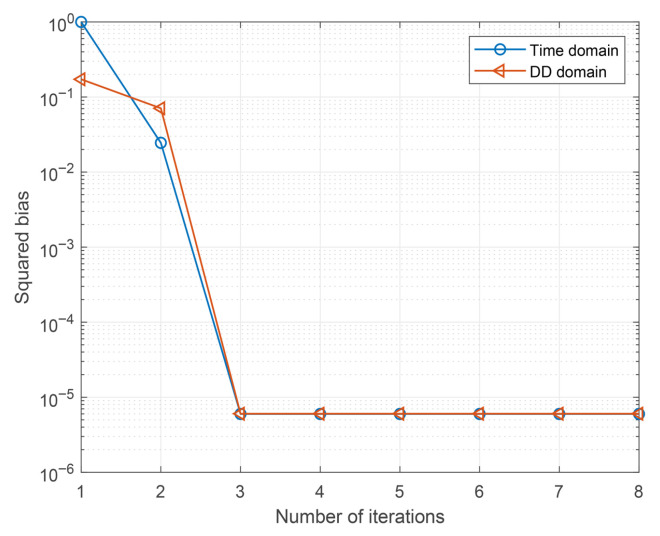
Evaluation of estimation bias for the proposed scheme.

Figure 4 illustrates the Monte Carlo simulation results of the squared estimation bias under a given channel realization, where the signal-to-noise ratio (SNR) is given by $E_{s}/N_{0}=12$ dB. The simulation parameters are considered as follows: P=4, M=32, N=16, QPSK, the channel coefficients of $\left[-0.27+0.35i,0.17+0.01i,0.56-0.33i,0.31-0.56i\right]$, the delay indices of $\left[0,1,4,2\right]$, and the Doppler indices of $\left[-1.40,2.98,-1.96,-3.66\right]$. The results are averaged over 1000 independent Monte Carlo trials, ensuring statistical stability of the estimated bias curves. As can be observed from the figure, the squared estimation biases of both the time-domain estimator and the DD-domain estimator decrease with the number of iterations, eventually converging to stable values. Moreover, the simulation results indicate that the residual estimation bias in both domains becomes negligible after sufficient iterations, thereby confirming the effectiveness and robustness of the proposed algorithm.

### 4.3. Variance Analysis by State Evolution

Recall that the above estimation bias analysis shows that the proposed estimators suffer from only negligible estimation bias in both domains. Thus, with the assumption of unbiased estimation, (46) can be rewritten as

(49) $${\mathrm{MSE}}_{T}\left(l\right)=\frac{1}{MN}\mathrm{Tr}\left(C_{m_{s}^{a,T}}\left(l\right)\right)=\frac{1}{MN}\mathrm{Tr}\left(C_{s}^{a,T}\left(l\right)\right),$$

where the MSE of the residual errors in the time domain is determined by the input a priori variance of the time-domain estimator. This also indicates that it is of great significance to track the evolution of the a priori variance of each domain, where the error performance of the proposed algorithm can be characterized by recursively calculating the asymptotic MSE performance.

Without loss of generality, we define the average a priori variance of the inputs to the time-domain estimator and the DD-domain estimator in the *l*-th iteration as

(50) $$v_{s}^{a,T}\left(l\right)\overset{\Delta }{=}\frac{1}{MN}\mathrm{Tr}\left(C_{s}^{a,T}\right)=\frac{1}{MN}\mathrm{Tr}\left(C_{s}^{e,\mathrm{DD}}\right)$$

and

(51) $$v_{s}^{a,\mathrm{DD}}\left(l\right)\overset{\Delta }{=}\frac{1}{MN}\mathrm{Tr}\left(C_{s}^{a,\mathrm{DD}}\right)=\frac{1}{MN}\mathrm{Tr}\left(C_{s}^{e,T}\right),$$

respectively. Under the unbiased estimation, the two states can be viewed as the asymptotically average MSEs of inputs in the *l*-th iteration. In the following, we will characterize the asymptotic MSE performance of the proposed detection scheme by state evolution.

Assume that the main diagonal entries of $C_{s}^{a,T}$ and $C_{s}^{a,\mathrm{DD}}$ are of the same value as $v_{s}^{a,T}\left(l\right)$ and $v_{s}^{a,\mathrm{DD}}\left(l\right)$, respectively, for the *l*-th iteration. $v_{s}^{a,\mathrm{DD}}\left(l\right)$ can be represented as [24]

(52) $$v_{s}^{a,\mathrm{DD}}\left(l\right)=\frac{1}{\frac{1}{v_{s}^{p,T}\left(l\right)}-\frac{1}{v_{s}^{a,T}\left(l\right)}},$$

according to (20). Substituting $C_{s}^{a,T}=v_{s}^{a,T}\left(l\right)I_{MN}$ into (19), the average a posteriori variance of $C_{s}^{p,T}$ can be obtained as

(53) $$\begin{array}{cc} v_{s}^{p,T}\left(l\right) & =v_{s}^{a,T}\left(l\right)-\frac{{\left(v_{s}^{a,T}\left(l\right)\right)}^{2}}{MN}\times \sum_{i=0}^{N-1}\mathrm{Tr}\left\{{\left(H_{A}^{i}\right)}^{H}\left(v_{s}^{a,T}\left(l\right)\right.H_{A}^{i}\right.\\ \; & \;\left.{\left.{\left(H_{A}^{i}\right)}^{H}+v_{s}^{a,T}\left(l\right)H_{B}^{i}{\left(H_{B}^{i}\right)}^{H}+N_{0}I_{2M}\right)}^{-1}H_{A}^{i}\right\} \end{array}$$

Based on (52) and (53), the state evolution from the state $v_{s}^{a,T}\left(l\right)$ to the state $v_{s}^{a,\mathrm{DD}}\left(l\right)$ can be obtained.

According to (34), the update of the state $v_{s}^{a,T}\left(l+1\right)$ can be given by

(54) $$v_{s}^{a,T}\left(l+1\right)=\frac{1}{\frac{1}{v_{s}^{p,\mathrm{DD}}\left(l\right)}-\frac{1}{v_{s}^{a,\mathrm{DD}}\left(l\right)}},$$

where

(55) $$v_{s}^{p,\mathrm{DD}}\left(l\right)\overset{\Delta }{=}\frac{1}{MN}\mathrm{Tr}\left(C_{s}^{p,\mathrm{DD}}\right)=\lim_{MN\to \infty }\frac{1}{MN}\mathrm{Tr}\left(C_{x}^{p,\mathrm{DD}}\right).$$

We define the average of the main diagonal entries of $C_{x}^{p,\mathrm{DD}}$ by [24]

(56) $$v_{x}^{p,\mathrm{DD}}\left(l\right)=E\left[{\left|x-E\left[x\left|x+\xi \right.\right]\right|}^{2}\right]=MSE\left(\eta_{\mathrm{DD}}\left(l\right)\right),$$

where *x* is an arbitrary DD-domain OTFS symbol and ξ is a complex AWGN sample with zero mean and variance $1/\eta_{\mathrm{DD}}$. In (56), $\eta_{\mathrm{DD}}\left(l\right)$ denotes the effective SNR for the DD domain in the *l*-th iteration, i.e., the ratio between the DD-domain OTFS signal energy and the average a priori variance of the inputs to the DD-domain estimator, given by

(57) $$\eta_{\mathrm{DD}}\left(l\right)=\frac{1}{v_{s}^{a,\mathrm{DD}}\left(l\right)},$$

where the DD-domain OTFS signal energy is normalized, i.e., $E\left[{\left|x\right|}^{2}\right]=1$. Thus, we have

(58) $$v_{s}^{p,\mathrm{DD}}\left(l\right)=v_{x}^{p,\mathrm{DD}}\left(l\right).$$

According to (54), (56), and (58), we can obtain the state evolution from the state $v_{s}^{a,\mathrm{DD}}\left(l\right)$ to the state $v_{s}^{a,T}\left(l+1\right)$. In the above state evolution, we use the Monte Carlo method to obtain $MSE\left(\eta_{\mathrm{DD}}\left(l\right)\right)$.

By iteratively updating the MSE state according to (52) and (54), the state evolution can then be derived.

### 4.4. MSE Boundary Analysis

In addition to the derived state evolution, we further apply bounding techniques to discuss the insights of the proposed scheme. Note that the proposed scheme adopts the SIC for time-domain estimation. Therefore, depending on whether SIC can fully eliminate the interference, the MSE performance can be bounded by applying the treating-interference-as-noise (TIN) strategy (corresponding to the worst-case scenario) and the genie-aided strategy (corresponding to the best-case scenario).

#### 4.4.1. TIN Strategy

TIN is a known interference management technique when the interference is sufficiently weak. Thus, TIN application corresponds to the worst-case scenario of the proposed scheme, where the interference is treated as noise regardless of its strength. Using the TIN strategy, the block-wise LMMSE estimation matrix $W_{\mathrm{MMSE}}^{i}$ for the *i*-th transmitted block $s_{i}$ becomes

(59) $$\begin{array}{cc} W_{\mathrm{MMSE}}^{i} & =C_{s_{i}}^{a,T}{\left(H_{A}^{i}\right)}^{H}{\left(H_{A}^{i}C_{s_{i}}^{a,T}{\left(H_{A}^{i}\right)}^{H}+\lambda_{\mathrm{ISI}}^{i}I_{2M}+N_{0}I_{2M}\right)}^{-1} \end{array}$$

where $\lambda_{\mathrm{ISI}}^{i}=\frac{1}{2M}\mathrm{Tr}\left(H_{B}^{i}C_{{\tilde{s}}_{i}}^{a,T}{\left(H_{B}^{i}\right)}^{H}\right)$ denotes the average interference energy of the *i*-th block. Correspondingly, the a posteriori estimation output $m_{s_{i}}^{p,T}$ of $s_{i}$ is replaced by

(60) $$m_{s_{i}}^{p,T}=m_{s_{i}}^{a,T}+W_{\mathrm{MMSE}}^{i}\left({\tilde{r}}_{i}-H_{A}^{i}m_{s_{i}}^{a,T}\right).$$

The a posteriori covariance matrix $C_{s_{i}}^{p,T}$, the extrinsic covariance matrix $C_{s_{i}}^{e,T}$, and the extrinsic mean $m_{s_{i}}^{e,T}$ of $s_{i}$ can still be calculated by (19), (20), and (21), respectively. The remaining steps are the same as those shown in Algorithm 1. In the state evolution, the key term $v_{s}^{p,T}\;\left(l\right)$ is modified by

(61) $$\begin{array}{cc} v_{s}^{p,T}\left(l\right) & =v_{s}^{a,T}\left(l\right)-\frac{{\left(v_{s}^{a,T}\left(l\right)\right)}^{2}}{MN}\times \sum_{i=0}^{N-1}\mathrm{Tr}\left\{{\left(H_{A}^{i}\right)}^{H}\left(v_{s}^{a,T}\left(l\right)\right.H_{A}^{i}\right.\\ \; & \;\left.{\left.{\left(H_{A}^{i}\right)}^{H}+\lambda_{\mathrm{ISI}}^{i}I_{2M}+N_{0}I_{2M}\right)}^{-1}H_{A}^{i}\right\} \end{array}$$

Based on (52) and (54), the average MSE via state evolution for the TIN case is thereby obtained.

#### 4.4.2. Genie-Aided Strategy

This strategy assumes no inter-block interference in the time domain. We use this baseline as the upper bound of the MSE performance in our analysis. The block-wise LMMSE estimation matrix $W_{\mathrm{MMSE}}^{i}$ for the *i*-th transmitted block $s_{i}$ is changed to

(62) $$W_{\mathrm{MMSE}}^{i}=C_{s_{i}}^{a,T}{\left(H_{A}^{i}\right)}^{H}{\left(H_{A}^{i}C_{s_{i}}^{a,T}{\left(H_{A}^{i}\right)}^{H}+N_{0}I_{2M}\right)}^{-1}.$$

The corresponding a posterior mean $m_{s_{i}}^{p,T}$ and covariance matrix $C_{s_{i}}^{p,T}$ are calculated by (60) and (19), respectively. The extrinsic mean $m_{s_{i}}^{e,T}$ and covariance matrix $C_{s_{i}}^{e,T}$ and the extrinsic of $s_{i}$ are calculated by (20) and (21), respectively. The key term $v_{s}^{p,T}\;\left(l\right)$ is modified by

(63) $$\begin{array}{cc} v_{s}^{p,T}\left(l\right) & =v_{s}^{a,T}\;\left(l\right)-\frac{{\left(v_{s}^{a,T}\left(l\right)\right)}^{2}}{MN}\times \sum_{i=0}^{N-1}\mathrm{Tr}\left\{{\left(H_{A}^{i}\right)}^{H}\left(v_{s}^{a,T}\left(l\right)\right.H_{A}^{i}\right.\\ \; & \;\left.{\left.{\left(H_{A}^{i}\right)}^{H}+N_{0}I_{2M}\right)}^{-1}H_{A}^{i}\right\}. \end{array}$$

Therefore, according to Algorithm 1 and the above variance analysis via state evolution, we can achieve an upper bound on the MSE performance under the genie-aided strategy.

It should be noted that both TIN and genie-aided bounds are of theoretical significance, as they together indicate whether the residual interference in the time domain can be minimized by the cross-domain iteration. As we will demonstrate in the numerical results part, the TIN bound and genie-aided bound will converge to each other after a sufficient number of cross-domain iterations. This suggests that the adopted reduced-size LMMSE estimator aligns well with the mechanism of the cross-domain iterative detection, where only the interference caused by the delay needs to be considered for the time-domain estimation, while the interference caused by Doppler can be resolved naturally by the cross-domain iteration.

### 4.5. Converged Error Performance Analysis

In this subsection, we will investigate the converged error performance by focusing on the effective DD-domain SNR under the TIN and genie-aided strategies. Observing (61) and (63), we find that $G_{A}^{i}\overset{\Delta }{=}H_{A}^{i}{\left(H_{A}^{i}\right)}^{H}$ is a Hermitian matrix. Applying eigenvalue decomposition, we can obtain $G_{A}^{i}=U_{A}^{i}\Lambda_{A}^{i}{\left(U_{A}^{i}\right)}^{H}$, where $U_{A}^{i}$ is a unitary matrix and $\Lambda_{A}^{i}$ is a diagonal matrix containing the descending eigenvalues of $G_{A}^{i}$, i.e., $\Lambda_{A}^{i}=diag\left\{\lambda_{A}^{i,0},\ldots ,\lambda_{A}^{i,j},\ldots ,\lambda_{A}^{i,2M-1}\right\}$. Note that $G_{A}^{i}$ has only *M* non-zero eigenvalues. Thus, we have the following Lemma.

**Lemma 1**  (Time-Domain a Posteriori Variance)**.** *The time-domain a posteriori variance $v_{s}^{p,T}\;\left(l\right)$ under the TIN strategy can be simplified by*

(64) $$v_{s}^{p,T}\left(l\right)=v_{s}^{a,T}\left(l\right)-\frac{v_{s}^{a,T}\left(l\right)}{MN}\sum_{i=0}^{N-1}\sum_{j=0}^{M-1}\frac{v_{s}^{a,T}\left(l\right)\lambda_{A}^{i,j}}{v_{s}^{a,T}\left(l\right)\lambda_{A}^{i,j}+\lambda_{\mathrm{ISI}}^{i}+N_{0}}.$$

*Similarly, let $\lambda_{\mathrm{ISI}}^{i}=0$. The time-domain a posteriori variance $v_{s}^{p,T}\;\left(l\right)$ under the genie-aided strategy can be obtained, i.e.,*

(65) $$v_{s}^{p,T}\left(l\right)=v_{s}^{a,T}\left(l\right)-\frac{v_{s}^{a,T}\left(l\right)}{MN}\sum_{i=0}^{N-1}\sum_{j=0}^{M-1}\frac{v_{s}^{a,T}\left(l\right)\lambda_{A}^{i,j}}{v_{s}^{a,T}\left(l\right)\lambda_{A}^{i,j}+N_{0}}.$$

**Proof.**  The proof is given in Appendix A. □

In order to obtain further insights, we apply Jensen’s inequality and the assumption that different resolvable paths have different delay indices, i.e., $l_{i}\neq l_{j}$, ∀i≠j, 1≤j,j≤P. (This assumption is realistic due to the fact that resolvable paths usually originate from geographically distinct reflectors. Thus, different resolvable paths are generally unlikely to have the exact same delay [30]. In particular, in sparse reflector environments, it is even less likely that different paths share the same delay [24].) We can derive the lower bound of the DD-domain a priori variance $v_{s}^{a,\mathrm{DD}}\left(l\right)$, as shown in the following Theorem.

**Theorem 1**  (Lower Bound of $v_{s}^{a,\mathrm{DD}}\left(l\right)$)**.** *The DD-domain a priori variance $v_{s}^{a,\mathrm{DD}}\left(l\right)$ under the TIN strategy is lower-bounded by*

(66) $$v_{s}^{a,\mathrm{DD}}\left(l\right)\geq \frac{\frac{v_{s}^{a,T}\left(l\right)}{2}{∥h∥}^{2}+N_{0}}{{∥h∥}^{2}},$$

*where $\lambda_{\mathrm{ISI}}^{i}=\frac{v_{s}^{a,T}\left(l\right)}{2}{∥h∥}^{2}$ represents the energy of the interference terms; $h={\left[h_{1},\ldots ,h_{P}\right]}^{T}$ denotes the channel coefficients vector; and ${∥\cdot ∥}^{2}$ denotes the square of the Euclidean norm of a vector.*

*Similarly, for the genie-aided strategy, we have*

(67) $$v_{s}^{a,\mathrm{DD}}\left(l\right)\geq \frac{N_{0}}{{∥h∥}^{2}}.$$

**Proof.**  The proof is given in Appendix B. □

Based on the above Theorem 1, we can obtain the upper bound of the DD-domain effective SNR $\eta_{\mathrm{DD}}\left(l\right)$, as shown in the following corollary.

**Corollary 1**  (The Upper Bound of $\eta_{\mathrm{DD}}\left(l\right)$ Under TIN Strategy)**.** *The DD-domain effective SNR $\eta_{\mathrm{DD}}\left(l\right)$ under the TIN strategy is upper-bounded by*

(68) $$\eta_{\mathrm{DD}}\left(l\right)\leq \frac{{∥h∥}^{2}}{\frac{v_{s}^{a,T}\left(l\right)}{2}{∥h∥}^{2}+N_{0}}.$$

**Proof.**  The proof can be derived from (57) and (66). □

**Corollary 2**  (The Upper Bound of $\eta_{\mathrm{DD}}\left(l\right)$ Under the Genie-Aided Strategy)**.** *The DD-domain effective SNR $\eta_{\mathrm{DD}}\left(l\right)$ under the genie-aided strategy is upper-bounded by*

(69) $$\eta_{\mathrm{DD}}\left(l\right)\leq \frac{{∥h∥}^{2}}{N_{0}}.$$

**Proof.**  The proof can be derived from (57) and (67). □

Compared with the TIN strategy, the difference in the genie-aided strategy is that there is no inter-block interference in the time domain, i.e., $\lambda_{\mathrm{ISI}}^{i}=0$ in (66). With the iteration between the time-domain detector and the DD-domain detector, $\lambda_{\mathrm{ISI}}^{i}$ gradually tends to 0. As shown in Corollaries 1 and 2, the upper bound of $\eta_{\mathrm{DD}}\left(l\right)$ under the TIN strategy converges to that under the genie-aided strategy with the iteration of the cross-domain detection. Furthermore, for a given fading channel, the effective DD-domain SNR of the proposed algorithm can theoretically approach the maximum receiver SNR with a sufficient number of iterations. This also indicates that the proposed algorithm can approach the error performance of MLSE theoretically given a sufficient number of iterations. In [24], the proposed cross-domain detection algorithm also approaches the maximum receiver SNR with sufficient iterations. This also shows that the proposed algorithm with low complexity has almost the same performance as that in [24] under sufficient iterations.

## 5. Numerical Results

We present the numerical results of the proposed schemes in this section. As an example, consider P=4, M=64, N=32, QPSK. The maximum delay and Doppler index are 10 and 5. In the figures, “Proposed 1” refers to the cross-domain iterative detection scheme based on the reduced-size LMMSE, while “Proposed 2” denotes the cross-domain iterative detection scheme employing the EVD-based reduced-size LMMSE. For comparison, “Full-size” corresponds to the cross-domain iterative detection scheme based on the full-size LMMSE proposed in [24].

**Figure 5:**
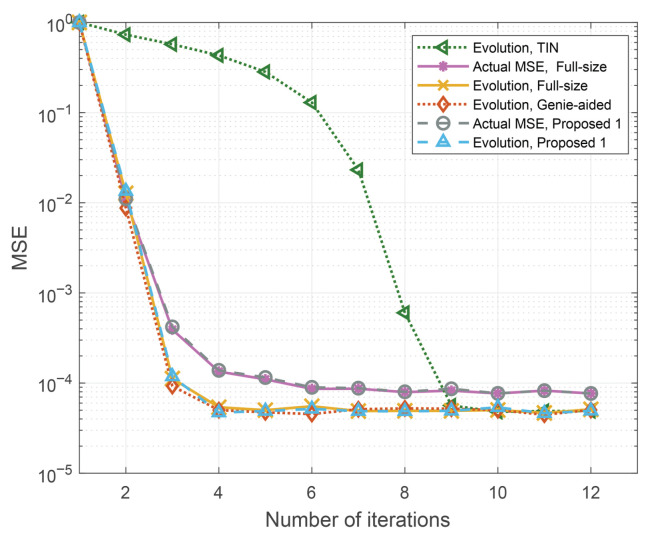
Comparison of time-domain state (MSE) evolution performance of the proposed scheme and the scheme in [24], as well as the derived bounds.

The state (MSE) evolution performance of the proposed scheme at a signal-to-noise ratio (SNR) $E_{s}/N_{0}=12$ dB is given in Figure 5, where both the actual MSE, the MSE evolution results, and the derived TIN and genie-aided bounds are presented in comparison to the results obtained from [24]. In the simulation, the channel coefficients, Doppler indices, and delay indices are $\left[-0.02-0.09i,0.40+0.73i,0.03+0.45i,0.15-0.43i\right]$, $\left[4.82,-3.23,1.38,-2.47\right]$, and $\left[0,8,4,6\right]$, respectively. As shown in Figure 5, our derived state evolution provides a good prediction of the actual MSE performance, where both the actual MSE and the MSE derived from the state evolution decrease first and then saturate at MSEs around $9\times 10^{-5}$ after sufficient iterations. Furthermore, we can observe that the derived state evolution matches perfectly with both the TIN and genie-aided bounds, which verifies the correctness of our derivation. Finally, we notice that the proposed scheme only exhibits marginal MSE loss compared to the scheme in [24] in early iterations both numerically and theoretically, and this loss becomes negligible with an increased number of iterations.

**Figure 6:**
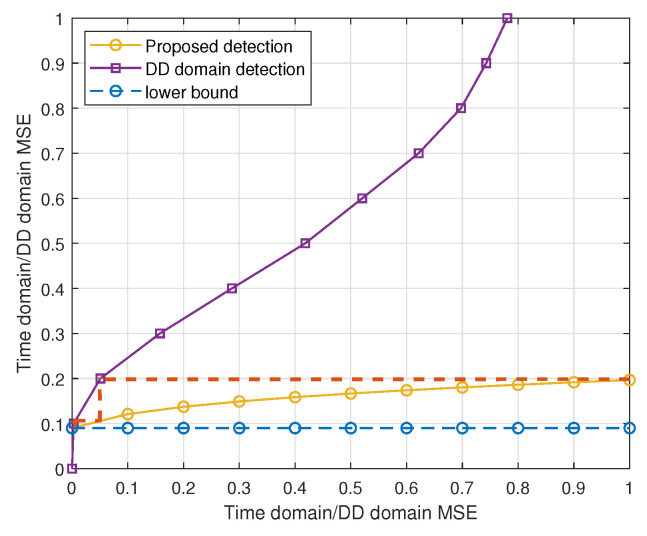
State (MSE) evolution performance and convergence trajectory of the proposed scheme. The red dashed line represents the convergence trajectory of the proposed detector.

Building upon the previous discussion of the time-domain state evolution in Figure 5, we now turn our attention to the DD domain. Figure 6 presents the state evolution of the proposed scheme (“proposed 1”), as well as its convergence trajectory, under the same channel parameters as in Figure 5 but at an SNR of $E_{s}/N_{0}=10$ dB. For clarity, the figure also includes the DD-domain error-state lower bound corresponding to the genie-aided strategy, as established in Theorem 1. In Figure 6, the horizontal and vertical axes represent the pairs of input–output MSEs under unbiased estimation in either the time or DD domain. The results demonstrate that the error states of the proposed scheme match well with the derived genie-aided lower bound, thereby confirming the validity of both our theoretical derivation and the proposed state evolution framework. Moreover, Figure 6 also shows the convergence trajectory of the proposed detector (depicted as the unlabeled dashed line). As shown, the algorithm converges to a stable MSE of approximately 0.0899 within three or four iterations. In general, under practical SNR ranges of 5–15 dB, the algorithm typically converges within three to five iterations. It is also worth noting that, at higher receive SNRs, the convergence point further decreases, leading to significantly lower MSE levels. This highlights the fact that the proposed scheme not only tracks the genie-aided bound but also approaches near-optimal detection performance under sufficiently high SNR conditions, thereby providing a favorable trade-off between complexity and performance.

**Figure 7:**
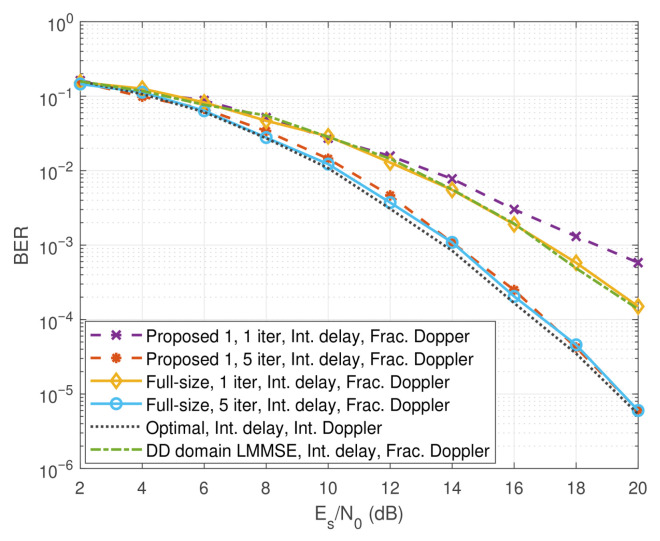
Comparison of BER performance of the proposed scheme (“Proposed 1”), the scheme in [24] (“Full-size”), and the optimal detection in [19] (“Optimal”).

In Figure 7, we compare the bit error rate (BER) performance of the proposed schemes with that of the full-size LMMSE baseline [24]. In the simulations, we consider integer delay and fractional Doppler, as fractional Doppler better reflects practical high-mobility scenarios. As observed from the figure, Proposed 1 suffers from a noticeable performance degradation with one iteration compared to the scheme in [24] at high SNRs due to the imperfect SIC adopted in the scheme in the early iterations. However, we notice that Proposed 1 shows roughly the same performance as the scheme in [24] with five iterations, and both their results converge to the optimal performance obtained by using the MP algorithm [19] with only integer delay and Doppler indices. Therefore, we observe that Proposed 1 enjoys a near-optimal performance with reduced complexity. Specifically, the proposed cross-domain iterative detection scheme based on the reduced-size LMMSE achieves approximately a 99% reduction in computational complexity compared with the conventional full-size LMMSE, while maintaining comparable detection performance. This demonstrates a favorable trade-off between computational efficiency and performance.

**Figure 8:**
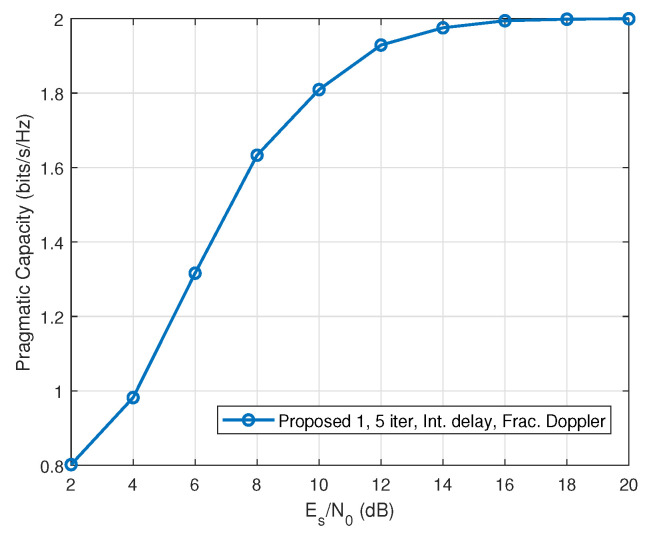
The pragmatic capacity of the proposed scheme (“Proposed 1”).

In addition to the BER performance shown in Figure 7, we evaluate the pragmatic capacity of the proposed reduced-size LMMSE scheme for QPSK signaling. Figure 8 presents the pragmatic capacity after five iterations. As observed, the capacity increases with SNR and approaches the theoretical maximum of 2 bits/s/Hz at a high SNR, indicating that the proposed algorithm not only achieves near-optimal detection performance but also maintains high spectral efficiency. This further highlights the practical benefits of the reduced computational complexity, enabling efficient real-time implementation without compromising throughput.

**Figure 9:**
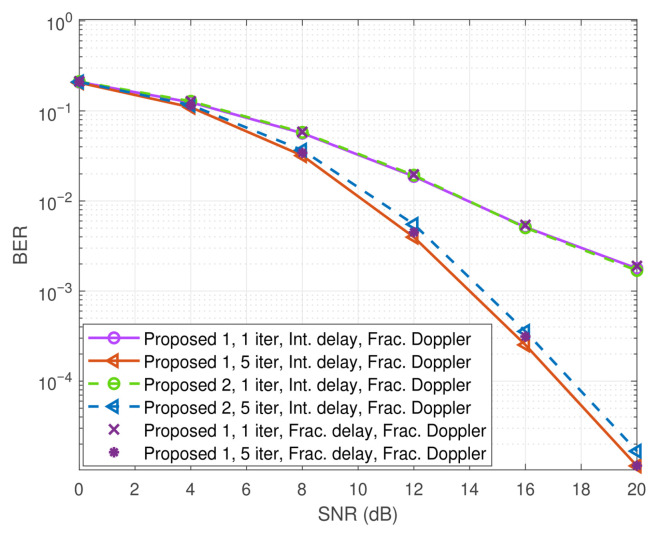
Comparison of BER performance of the proposed schemes.

Figure 9 further illustrates the performance of the two proposed schemes, where the parameters are set to M=32 and N=16. It can be observed that, as the SNR increases, Proposed 2 exhibits inferior performance compared with Proposed 1. This performance degradation arises from two main factors. On the one hand, the EVD method relies on the asymptotic assumption that MN→∞, which is not fully satisfied in the finite-size simulation setting. On the other hand, the computation of extrinsic information involves inversion, which is prone to numerical instability, particularly in high-SNR regimes. Nevertheless, the observed performance loss remains acceptable when considering the overall computational complexity. In addition, Figure 9 also presents a BER performance comparison of Proposed 1 under fractional delay against its performance under integer delay. The results show that the proposed scheme continues to perform effectively under fractional delay, highlighting its effectiveness, robustness, and applicability in more general and practical channel conditions.

## 6. Conclusions

In this paper, we studied OTFS transmission using the discrete Zak transform and derived vectorized input–output relations in both the time and delay–Doppler domains, revealing that delay and Doppler effects can be decoupled. Based on this, we proposed a reduced-complexity cross-domain iterative detection algorithm that combines a reduced-size LMMSE estimator in the time domain with symbol-by-symbol detection in the DD domain, where extrinsic information is exchanged via a unitary transformation. By applying eigenvalue decomposition, the LMMSE complexity becomes independent of the number of iterations. Analytical and numerical results show that the estimator bias is negligible, the TIN and genie-aided bounds converge with sufficient iterations, and the effective DD-domain SNR approaches the maximum receiver SNR. Overall, the proposed scheme achieves near-optimal error performance with reduced computational complexity.

## Data Availability

The data presented in this study are available on request from the corresponding author.

## Appendix A. Derivation for (64)

According to (61) and $\mathrm{Tr}\left(\mathrm{AB}\right)=\mathrm{Tr}\left(\mathrm{BA}\right)$, we have

(A1) $$\begin{array}{cc} v_{s}^{p,T}\left(l\right) & =v_{s}^{a,T}\left(l\right)-\frac{{\left(v_{s}^{a,T}\left(l\right)\right)}^{2}}{MN}\sum_{i=0}^{N-1}\mathrm{Tr}\left\{\left(\left(v_{s}^{a,T}\left(l\right)\right.H_{A}^{i}\right.\right.\\ \; & \;+{\left(H_{A}^{i}\right)}^{H}{\left.\lambda_{\mathrm{ISI}}^{i}I_{2M}+N_{0}I_{2M}\right)}^{-1}\left.H_{A}^{i}{\left(H_{A}^{i}\right)}^{H}\right\}. \end{array}$$

Substituting $H_{A}^{i}{\left(H_{A}^{i}\right)}^{H}=U_{A}^{i}\Lambda_{A}^{i}{\left(U_{A}^{i}\right)}^{H}$ into (A1), we get (A2), i.e.,

(A2) $$\begin{array}{cc} v_{s}^{p,T}\left(l\right)\; & =v_{s}^{a,T}\left(l\right)-\frac{{\left(v_{s}^{a,T}\left(l\right)\right)}^{2}}{MN}\sum_{i=0}^{N-1}\mathrm{Tr}\left\{{\left(\left(v_{s}^{a,T}\left(l\right)\right.U_{A}^{i}\Lambda_{A}^{i}{\left(U_{A}^{i}\right)}^{H}+\lambda_{\mathrm{ISI}}^{i}I_{2M}+N_{0}I_{2M}\right)}^{-1}\right.\\ \; & \;\left.U_{A}^{i}\Lambda_{A}^{i}{\left(U_{A}^{i}\right)}^{H}\right\}\\ \; & =v_{s}^{a,T}\left(l\right)-\frac{{\left(v_{s}^{a,T}\left(l\right)\right)}^{2}}{MN}\sum_{i=0}^{N-1}\mathrm{Tr}\left\{{\left({\left(U_{A}^{i}\right)}^{H}\right)}^{-1}{\left(\left(v_{s}^{a,T}\left(l\right)\right.\Lambda_{A}^{i}+\lambda_{\mathrm{ISI}}^{i}I_{2M}+N_{0}I_{2M}\right)}^{-1}\right.\\ \; & \;\left.{\left(U_{A}^{i}\right)}^{-1}U_{A}^{i}\Lambda_{A}^{i}{\left(U_{A}^{i}\right)}^{H}\right\}\\ \; & =v_{s}^{a,T}\left(l\right)-\frac{{\left(v_{s}^{a,T}\left(l\right)\right)}^{2}}{MN}\sum_{i=0}^{N-1}\mathrm{Tr}\left\{{\left(\left(v_{s}^{a,T}\left(l\right)\right.\Lambda_{A}^{i}+\lambda_{\mathrm{ISI}}^{i}I_{2M}+N_{0}I_{2M}\right)}^{-1}\Lambda_{A}^{i}\right\}\\ \; & =v_{s}^{a,T}\left(l\right)-\frac{v_{s}^{a,T}\left(l\right)}{MN}\sum_{i=0}^{N-1}\sum_{j=0}^{M-1}\frac{v_{s}^{a,T}\left(l\right)\lambda_{A}^{i,j}}{v_{s}^{a,T}\left(l\right)\lambda_{A}^{i,j}+\lambda_{\mathrm{ISI}}^{i}+N_{0}}. \end{array}$$

This completes the proof of Lemma 1.

## Appendix B. Derivation for (66)

Observing (64), we define a function, i.e., $f\left(\lambda \right)=\frac{v\lambda }{v\lambda +\eta }$, with respect to λ, where v≥0 and η>0. Since its second-order derivative, i.e., $f^{\prime \prime }\left(\lambda \right)=-\frac{2v^{2}\eta }{{\left(v\lambda +\eta \right)}^{3}}$, is always negative, the considered function is concave. According to Jensen’s inequality, (A2) can be represented as

(A3) $$v_{s}^{p,T}\left(l\right)\geq v_{s}^{a,T}\left(l\right)-\frac{v_{s}^{a,T}\left(l\right)}{N}\sum_{i=0}^{N-1}\frac{\frac{v_{s}^{a,T}\left(l\right)}{M}\sum_{j=0}^{M-1}\lambda_{A}^{i,j}}{\frac{v_{s}^{a,T}\left(l\right)}{M}\sum_{j=0}^{M-1}\lambda_{A}^{i,j}+\lambda_{\mathrm{ISI}}^{i}+N_{0}},$$

where the lower bound becomes tighter with the decrease in $v_{s}^{a,T}\left(l\right)$ and the equality is achieved when $v_{s}^{a,T}\left(l\right)=0$. Note that $\sum_{j=0}^{M-1}\lambda_{A}^{i,j}=M{∥h∥}^{2}$ and $\lambda_{\mathrm{ISI}}^{i}=\mathrm{Tr}\left(H_{B}^{i}C_{{\tilde{s}}_{i}}^{a,T}{\left(H_{B}^{i}\right)}^{H}\right)/\left(2M\right)=v_{s}^{a,T}\left(l\right){∥h∥}^{2}/2$, $\forall i\in \left\{0,1,\ldots ,N-1\right\}$, where $h={\left[h_{1},\ldots ,h_{P}\right]}^{T}$ denotes the channel coefficients vector and ${∥\cdot ∥}^{2}$ denotes the square of the Euclidean norm of a vector. Thus, (A3) is rewritten as

(A4) $$\begin{array}{cc} v_{s}^{p,T}\left(l\right) & \geq v_{s}^{a,T}\left(l\right)-\frac{v_{s}^{a,T}\left(l\right)}{N}\sum_{i=0}^{N-1}\frac{v_{s}^{a,T}\left(l\right){∥h∥}^{2}}{v_{s}^{a,T}\left(l\right){∥h∥}^{2}+\frac{v_{s}^{a,T}\left(l\right)}{2}{∥h∥}^{2}+N_{0}}\\ \; & =v_{s}^{a,T}\left(l\right)-\frac{{\left(v_{s}^{a,T}\left(l\right)\right)}^{2}{∥h∥}^{2}}{v_{s}^{a,T}\left(l\right){∥h∥}^{2}+\frac{v_{s}^{a,T}\left(l\right)}{2}{∥h∥}^{2}+N_{0}}. \end{array}$$

Substituting (A4) into (52), we arrive at

(A5) $$\begin{array}{cc} v_{s}^{a,\mathrm{DD}}\left(l\right) & =\frac{1}{\frac{1}{v_{s}^{p,T}\left(l\right)}-\frac{1}{v_{s}^{a,T}\left(l\right)}}\\ \; & \geq \frac{1}{\frac{1}{v_{s}^{a,T}\left(l\right)-\frac{{\left(v_{s}^{a,T}\left(l\right)\right)}^{2}{∥h∥}^{2}}{v_{s}^{a,T}\left(l\right){∥h∥}^{2}+\frac{v_{s}^{a,T}\left(l\right)}{2}{∥h∥}^{2}+N_{0}}}-\frac{1}{v_{s}^{a,T}\left(l\right)}}\\ \; & =\frac{v_{s}^{a,T}\left(l\right)-\frac{{\left(v_{s}^{a,T}\left(l\right)\right)}^{2}{∥h∥}^{2}}{v_{s}^{a,T}\left(l\right){∥h∥}^{2}+\frac{v_{s}^{a,T}\left(l\right)}{2}{∥h∥}^{2}+N_{0}}}{\frac{v_{s}^{a,T}\left(l\right){∥h∥}^{2}}{v_{s}^{a,T}\left(l\right){∥h∥}^{2}+\frac{v_{s}^{a,T}\left(l\right)}{2}{∥h∥}^{2}+N_{0}}}\\ \; & =\frac{\frac{v_{s}^{a,T}\left(l\right)}{2}{∥h∥}^{2}+N_{0}}{{∥h∥}^{2}}. \end{array}$$

This completes the proof of Theorem 1.
